# Delay chemical master equation: direct and closed-form solutions

**DOI:** 10.1098/rspa.2015.0049

**Published:** 2015-07-08

**Authors:** Andre Leier, Tatiana T. Marquez-Lago

**Affiliations:** 1 Okinawa Institute of Science and Technology, Onna-son, Okinawa, Japan; 2 Integrative Systems Biology Unit, Okinawa Institute of Science and Technology, Onna-son, Okinawa, Japan

**Keywords:** delay chemical master equation, delay stochastic simulation algorithm, direct solution, closed-form solution

## Abstract

The stochastic simulation algorithm (SSA) describes the time evolution of a discrete nonlinear Markov process. This stochastic process has a probability density function that is the solution of a differential equation, commonly known as the chemical master equation (CME) or forward-Kolmogorov equation. In the same way that the CME gives rise to the SSA, and trajectories of the latter are exact with respect to the former, trajectories obtained from a delay SSA are exact representations of the underlying delay CME (DCME). However, in contrast to the CME, no closed-form solutions have so far been derived for any kind of DCME. In this paper, we describe for the first time direct and closed solutions of the DCME for simple reaction schemes, such as a single-delayed unimolecular reaction as well as chemical reactions for transcription and translation with delayed mRNA maturation. We also discuss the conditions that have to be met such that such solutions can be derived.

## 1. Introduction

The Markov jump formalism has been widely used to describe the stochastic nature of chemical reactions [1,2], gene regulation [3] and other systems involving randomly fluctuating population sizes [4]. In the terminology of chemical reactions, the number of molecules of all present chemical species determines the state of the system, and the system dynamics are governed by a set of reactions involving these species. Specifically, this can be modelled as a continuous-time, discrete space Markov process and represented by a system of ordinary differential equations, the so-called chemical master equation (CME), describing the temporal evolution of the probability distribution over all possible states of the system.

The CME can be directly, analytically solved only for very simple, linear systems [5]. In some cases, approximate numerical solutions are possible by truncating the state space [6,7], but when the probability mass is distributed over a very large number of states this task can still be computationally infeasible. Alternatively, one can use sampling methods such as the stochastic simulation algorithm (SSA) [1], which generates trajectories in the state space that are exact realizations of the Markov process.

Rather recently, the CME framework has been extended by the concept of delays, to a delay CME, leading to the acronym DCME [3]. For certain biochemical models such as gene transcription and translation, it has been shown that delays become necessary to describe the system dynamics more accurately. In order to deal with time delays in discrete stochastic systems Barrio *et al.* [3] proposed a delay SSA (DSSA) and the corresponding master equation formulation.

Essentially, in chemical reaction kinetics, delays are used to lump complex processes that often consist of many chemical reactions and species, or even represent diffusion processes [3,8]. That is, instead of modelling every single detail of a chemical or diffusion process, a task that is quite computationally intensive, a delayed reaction is used to describe and mimic the effects of these processes on the overall system dynamics. For instance, delays are used to model transcription and translation processes without including any underlying mechanisms, such as each movement of RNA polymerase along the DNA strand, or decoding of mRNA by the ribosomal machinery [3]. Diffusion from the plasma membrane to the nucleus (and vice versa) can also be accurately modelled in a purely temporal manner, by introducing a transport reaction with an associated delay distribution [8].

By incorporating all relevant information into a delayed model, computational simulations of relevant biochemical processes that would otherwise be computationally prohibitive can be performed. Likewise, exact model reduction methodologies have been developed, through the appropriate use of delays [9,10].

Hence, a better understanding of the DCME becomes essential. For that, and to ease readability throughout this article, we will first introduce the different algorithms covering delay stochastic kinetics. We will then define a simple DCME, followed by a general DCME framework covering all possible chemical kinetics scenarios. From here, we will show how an exact solution can be derived in certain cases, and also portray cases in which the DCME can be equivalently solved by a CME with time-varying parameters. These two observations have never been described in the literature before, opening up both applications and alternative methodologies to solve stochastic chemical kinetics with prescribed delays.

## 2. Delay stochastic simulation algorithms

In recent years, several DSSA implementations have been proposed. The first DSSA algorithm was presented in [11]. Albeit helpful, this approach had a couple of flaws: (i) it ignored waiting times for delayed reactions and (ii) the update of both reactant and product species involved in a delayed reaction always happened at the end of the time delay. The latter aspect causes delayed reactions to be initiated for the very same reactants over and over again, which may not reflect the biochemical reality. For instance, the authors in [11] allowed a protein to start a process of delayed degradation after it had already initiated such a degradation process (and was still undergoing this process). This has been shown to cause artificial oscillatory dynamics in the protein levels [12]. Hence, this algorithm is usually not considered accurate.

Independently, Barrio *et al*. [3] developed the first exact DSSA, where the concept of non-consuming and consuming reactions was introduced. In short, when a non-consuming reaction occurs, the numbers of all its reactant molecules remain unchanged (as in [11]). However, for consuming reactions, the numbers of reactant molecules are updated at the time of initiation while numbers of product molecules are updated at the end of the time delay. The choice of the reaction type, consuming or non-consuming, strongly depends on the biochemical context. For instance, a transport process should be defined by a consuming reaction: a molecule physically leaves a compartment and appears at a different location after some time, implying updates at the initiation of the reaction and at the end of the delay. Transcription and translation processes, on the other hand, can be defined as non-consuming reactions: a single gene is transcribed simultaneously (by several RNA polymerases) and the DNA itself is not consumed by the first transcription. The algorithm in [3] was later termed ‘rejection algorithm’ because of the way it deals with the update of delayed reactions [13]. Here it was also confirmed that the rejection method is fully accurate, as is also the approach in [13].

Of note, it is the very distinction between consuming and non-consuming reactions that enables exact DSSAs to accurately represent biochemical processes. Likewise, it is these definitions that allow for the analytical description of a DCME, as will be explained in the following section. Thus, it is important to define delayed reactions properly with respect to their update points (consuming versus non-consuming), as different classifications may yield different simulation results.

The second exact DSSA [13], termed the ‘direct method’, avoids reaction rejections by calculating the piecewise probability density function (PDF) for the next reaction to appear in any of the time intervals $[T_{0},T_{1}),[T_{1},T_{2}),\ldots ,[T_{k-1},T_{k}),[T_{k},+\infty ),$ given *k* update time points *T*_*i*_ for delayed reactions that had been initiated in the past (and by defining *T*_0_=*t*). This PDF is defined for each distinct interval, since the propensity functions are piecewise constant (i.e. they only change at every update point *T*_*i*_). Then, the interval [*T*_*i*_,*T*_*i*+1_) in which the next reaction is about to occur is obtained by finding the index *i* such that

$$\sum_{j=1}^{i}(T_{j}-T_{j-1})\;a_{0}\;(\text{X}(T_{j-1}))\leq \ln\left(\frac{1}{r}\right)<\sum_{j=1}^{i+1}(T_{j}-T_{j-1})\;a_{0}\;(X(T_{j-1})),$$

for *r*∈**U**(0,1), and *a*_0_(***X***(*t*)) being the sum of all reaction propensities for the system state ***X***(*t*) at time *t*. The direct method updates the system state according to the delayed reactions that are due at update time points *T*_1_,…*T*_*i*_ and advances the time to

$$t=T_{i}+\frac{\ln\;(1/r)-\sum_{j=1}^{i}(T_{j}-T_{j-1})\;a_{0}\;(\text{X}(T_{j-1}))}{a_{0}\;(\text{X}(T_{i}))}.$$

It has been argued that this algorithm is faster as it does not waste random numbers. However, calculating the correct piecewise PDF also entails computational costs. Thus, the performance comparison of the two algorithms likely depends on each system under investigation, including the number of update points during simulation time and other factors.

It is also worth noting the direct method is closely related to simulation methods for reaction systems with time-dependent propensity functions [14,15]. In [14], a modified next reaction method for time-dependent propensities and time delays was proposed. In similarity to the SSA next reaction method, each reaction has a putative reaction time, but here it also includes update time points of delayed reactions. The next reaction to be either executed (if it is non-delayed) or initiated or updated (for delayed reactions) is always the one with the shortest putative reaction time.

## 3. The delay chemical master equation

Assume that the given chemical reaction system contains *N* molecular species $S=\{S_{1},\ldots ,S_{N}\}.$ Let *X*_*i*_(*t*) denote the number of species *S*_*i*_ at time *t*. Then the vector ***X***(*t*)=(*X*_1_(*t*),…,*X*_*N*_(*t*)) describes the system's state at time *t*. As is also the case for the CME, the DCME is valid under the assumption of well-mixedness of all chemical species.

Among *M* reactions, the first *M*_0d_ reactions are assumed to be non-delayed, *M*_dc_ reactions are delayed consuming reactions, and *M*_dn_ reactions are non-consuming delayed reactions. The corresponding sets of reactions are denoted with $R_{0d}$, $R_{\mathrm{dc}}$ and $R_{\mathrm{dn}},$ respectively. In addition, we define $R_{d}=R_{\mathrm{dc}}\cup R_{\mathrm{dn}}$ (the set of all delayed reactions) and $R=R_{d}\cup R_{0d}$ (the set of all reactions). The delay of a reaction $R_{j}\in R_{d}$ is denoted with *τ*_*j*_.

As explained in the previous section, non-delayed and delayed non-consuming reactions have only one update point (for updating both reactant and product molecule numbers): the former when the non-delayed reaction happens, the latter when the delay finishes. Their corresponding stoichiometric (update) vectors are denoted with *ν*. Only delayed consuming reactions have two update points: at the time of initiation and the end of the delay. Here, we denote with *ν*^*r*^ the update vector at time of initiation for updating reactant molecule numbers while we denote with *ν*^*p*^ the update vector at the end of the delay for updating product molecule numbers. For each reaction $R_{j}\in R$, *a*_*j*_ denotes the corresponding propensity function.

Moreover, the system is assumed to be at time *t*_0_ in state ***X***(*t*_0_)=***X***_0_ and to have a history (memory) of *K* previously initiated but still unfinished (ongoing) reactions as described by the set $H_{0}=\{(R_{i,},T_{i})|R_{i}\in R_{d},T_{i}>t_{0}\;\forall \;i=1..K\},$ where reaction *R*_*i*_ is a delayed reaction that is due for completion at time *T*_*i*_.

At this stage, it is useful to recall that for a reaction system without delays the CME has the form

3.1 $$\frac{\partial }{\partial t}P(\text{X},t)=-\sum_{j=1}^{M_{0d}}a_{j}(\text{X})P(\text{X},t)+\sum_{j=1}^{M_{0d}}a_{j}(\text{X}-\nu_{j})P(\text{X}-\nu_{j},t),$$

where *P*(***X***,*t*)=*P*(***X***,*t*|***X***_0_,*t*_0_) is the conditional probability of finding the system in state ***X*** at time *t* provided it had been in the initial state ***X***_0_ at time *t*_0_. By using a similar notation, the DCME was first introduced in [3] and given as

3.2 $$\begin{array}{rl} \frac{\partial }{\partial t}P(\text{X},t) & =-\sum_{j=1}^{M_{0d}}a_{j}(\text{X})P(\text{X},t)+\sum_{j=1}^{M_{0d}}a_{j}(\text{X}-\nu_{j})P(\text{X}-\nu_{j},t)-\sum_{j=M_{0d}+1}^{M}\sum_{{\text{X}}_{i}\in I(\text{X})}a_{j}({\text{X}}_{i})P(\text{X},t;{\text{X}}_{i},t-\tau_{j})\\ & \;+\sum_{j=M_{0d}+1}^{M}\sum_{{\text{X}}_{i}\in I(\text{X})}a_{j}({\text{X}}_{i})P(\text{X}-\nu_{j},t;{\text{X}}_{i},t-\tau_{j}). \end{array}$$

Here, ***I***(***X***) represents the set of all possible system states in the past from which state ***X*** was able to follow (via a chain of chemical reactions). The probability $P(\text{ X},t)=P(\text{ X},t|{\text{ X}}_{0},t_{0},H_{0})$ is the conditional probability of finding the system in state ***X*** at time *t* provided it had been in the initial state ***X***_0_ at time *t*_0_ with initial history $H_{0}.$

The idea behind this formulation of the DCME is that, for a reaction *R*_*j*_ with delay *τ*_*j*_, the current system state should depend on the historical state at time *t*−*τ*_*j*_. Keeping this in mind, the third term on the right-hand side of the equation above can be interpreted as the probability that a reaction *R*_*j*_ occurred in [*t*−*τ*_*j*_,*t*−*τ*_*j*_+d*t*) that is about to be updated in [*t*,*t*+d*t*), which will push the system out of state ***X***. In the same context, the last term can be seen as the probability that the system is an update of reaction *R*_*j*_ away from state ***X*** and this update will happen in [*t*,*t*+d*t*).

This formulation has a problem, though. It does not distinguish between consuming and non-consuming reactions. To be more precise, it is a correct DCME but only for systems with non-delayed and delayed non-consuming reactions when the update happens only at the end of a time delay. The following scenario is not reflected in the equation: for systems with consuming reactions it is possible that such a reaction *R*_*j*_ is initiated in [*t*,*t*+d*t*) and the state update via $\nu_{j}^{r}$ brings the system from state $\text{ X}-\nu_{j}^{r}$ to state ***X***. Likewise, it is possible that such a reaction is triggered in [*t*,*t*+d*t*) and hence reduces the probability that the system remains in state ***X*** during the interval [*t*,*t*+d*t*).

Along those lines, in [16], it has been stated that the DCME in [3] is incorrect. However, this interpretation is wrong in that the description in [3] is just a special case of the general DCME. This probably stems from a misinterpretation of concepts (consuming versus non-consuming reactions). Furthermore, the work in [16] proposes two DSSAs, which incidentally are just special cases of the DSSA in [3]. Nevertheless, the work in [16] is quite useful in that it shows how specific DCMEs can be derived, and this in turn allowed us to derive the correct general expression.

In summary, by incorporating the above-mentioned scenarios into one formula, we obtain the following correct DCME, accounting for both consuming and non-consuming delay reactions:

3.3 $$\begin{array}{rl} \frac{\partial }{\partial t}P(\text{X},t) & =-\sum_{R_{j}\in M_{0d}}a_{j}(\text{X})P(\text{X},t)+\sum_{R_{j}\in M_{0d}}a_{j}(\text{X}-\nu_{j})P(\text{X}-\nu_{j},t)\\ & \;-\sum_{R_{j}\in M_{dn}}\sum_{{\text{X}}_{i}\in I(\text{X})}a_{j}({\text{X}}_{i})P(\text{X},t;{\text{X}}_{i},t-\tau_{j})+\sum_{R_{j}\in M_{dn}}\sum_{{\text{X}}_{i}\in I(\text{X})}a_{j}({\text{X}}_{i})P(\text{X}-\nu_{j},t;{\text{X}}_{i},t-\tau_{j})\\ & \;-\sum_{R_{j}\in M_{dc}}\sum_{{\text{X}}_{i}\in I(\text{X})}a_{j}({\text{X}}_{i})P(\text{X},t;{\text{X}}_{i},t-\tau_{j})+\sum_{R_{j}\in M_{dc}}\sum_{{\text{X}}_{i}\in I(\text{X})}a_{j}({\text{X}}_{i})P(\text{X}-\nu_{j}^{p},t;{\text{X}}_{i},t-\tau_{j})\\ & \;-\sum_{R_{j}\in M_{dc}}a_{j}(\text{X})P(\text{X},t)+\sum_{R_{j}\in M_{dc}}a_{j}(\text{X}-\nu_{j}^{r})P(\text{X}-\nu_{j}^{r},t). \end{array}$$

The first term refers to the probability that the system is in state ***X*** at time *t* while a non-delayed reaction *R*_*j*_ occurs, while the second term refers to the probability that the system is one non-delayed reaction *R*_*j*_ removed from state ***X*** and reaction *R*_*j*_ happens, yielding system state ***X***. The next term refers to the probability that the system is in state ***X*** at time *t* while a delayed non-consuming reaction *R*_*j*_ gets updated that had been previously triggered at time *t*−*τ*_*j*_. The fourth term corresponds to the opposite case, where the system is one update of *R*_*j*_ away from state ***X***, namely in ***X***−*ν*_*j*_, and the update due to happen yields state ***X***. The last four terms refer to the occurrence of a delayed consuming reaction *R*_*j*_. The fifth and sixth terms are equivalent to the third and fourth terms, with respect to state changes originating from the second update point (after the reaction finished). The last two terms, seventh and eighth, refer to the probability that the system is in state ***X*** at time *t* when *R*_*j*_ occurs and to the probability that the system is one reaction *R*_*j*_ removed from state ***X*** and reaction *R*_*j*_ is initiated, yielding system state ***X***.

Note that this most general expression is different to any previously published DCME. Even though the DCMEs in [16] come quite close to the general expression, they are, just like the DCME in [3], special cases.

We can now even go a step further and introduce distributed delays instead of constant delays. Then, equation (3.3) becomes

3.4 $$\begin{array}{rl} \frac{\partial }{\partial t}P(\text{X},t) & =-\sum_{R_{j}\in M_{0d}}a_{j}(\text{X})P(\text{X},t)+\sum_{R_{j}\in M_{0d}}a_{j}(\text{X}-\nu_{j})P(\text{X}-\nu_{j},t)\\ & \;-\sum_{R_{j}\in M_{\mathrm{dn}}}\sum_{{\text{X}}_{i}\in I(\text{X})}\int_{0}^{t}\tau_{j}(s)a_{j}({\text{X}}_{i})P(\text{X},t;{\text{X}}_{i},t-s)\;\text{d}s\\ & \;+\sum_{R_{j}\in M_{\mathrm{dn}}}\sum_{{\text{X}}_{i}\in I(\text{X})}\int_{0}^{t}\tau_{j}(s)a_{j}({\text{X}}_{i})P(\text{X}-\nu_{j},t;{\text{X}}_{i},t-s)\;\text{d}s\\ & \;-\sum_{R_{j}\in M_{\mathrm{dc}}}\sum_{{\text{X}}_{i}\in I(\text{X})}\int_{0}^{t}\tau_{j}(s)a_{j}({\text{X}}_{i})P(\text{X},t;{\text{X}}_{i},t-s)\;\text{d}s\\ & \;+\sum_{R_{j}\in M_{\mathrm{dc}}}\sum_{{\text{X}}_{i}\in I(\text{X})}\int_{0}^{t}\tau_{j}(s)a_{j}({\text{X}}_{i})P(\text{X}-\nu_{j}^{p},t;{\text{X}}_{i},t-s)\;\text{d}s\\ & \;-\sum_{R_{j}\in M_{\mathrm{dc}}}a_{j}(\text{X})P(\text{X},t)+\sum_{R_{j}\in M_{\mathrm{dc}}}a_{j}(\text{X}-\nu_{j}^{r})P(\text{X}-\nu_{j}^{r},t), \end{array}$$

where *τ*_*j*_ denotes the PDF of the delay distribution associated with reaction *R*_*j*_.

Importantly, while the CME describes a continuous-time Markov process (i.e. a memory-less process), this property no longer holds for the DCME. In other words, by introducing delays, the master equation loses its Markov property, since transitions from the current state to any future state no longer solely depend on the current state but also on reactions that have been triggered in the past. Indeed, any implementation of a DSSA necessarily requires storage of delayed reactions that have been triggered in the past and need to be updated in a future time point. This storage can be thought of as a memory of the process. Nonetheless, the apparently non-Markovian process described by the DCME could still have a Markovian representation. Here, typically the trick is to expand the description of the current and/or future state. Intuitively, one could include future update points in the description of current states. However, the expanded state space in this case is no longer countable and transitions cannot be represented with a transition rate matrix, a requirement for this process to be a continuous-time Markov process.

Finally, calculating the DCME is not straightforward even when using constant delays and for simplest cases, due to the very complicated probability terms and the sums over all possible previous system states ***X***_*i*_. In contrast to the CME, not a single example could be found in the literature where the DCME is explicitly calculated. In this paper, and for a special case, we solve the DCME directly for the first time.

## 4. Exact closed-form solution of a delay chemical master equation

**Figure 1.**
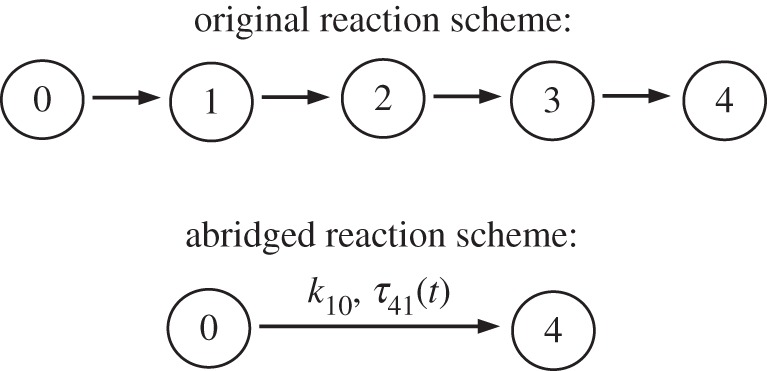
A simple linear five-state (species) reaction system and its abridged scheme.

So far, the DCME has only been studied by generating trajectories with a DSSA. Here, we show how it may be possible to actually solve and study the DCME by means of closed-form analytical solutions, for certain types of systems. To do so, let us consider the five-state system illustrated in figure 1.

As has been previously shown [9,10], the temporal dynamics of the number of molecules in state 0 and state 4 of the original linear reaction scheme can be described exactly by an abridged (reduced) reaction scheme that only involves states 0 and 4. The latter consists of a reaction with a waiting time identical to that of the original reaction 0→1 and an associated first-passage delay distribution *τ*_41_ describing the transition 1→4. As such, the abridged system can be easily simulated with a DSSA.

The delay distribution *τ*_41_(*t*) is simply the PDF of the sum of three random variables, each describing the time of a transition *j*→*j*+1 and having an exponential distribution with parameter *k*_*j*+1,*j*_( *j*=1,2,3). In other words, *τ*_41_(*t*) is the convolution of such exponential distributions, namely

4.1 $$\tau_{41}=\tau_{21}*\tau_{32}*\tau_{43},$$

where * denotes the convolution of the PDFs. At this stage, we introduce also the PDF for the first-passage time describing the transition 0→4, which, following the same argument, is

4.2 $$\tau_{40}=\tau_{10}*\tau_{41}.$$

For convenience, let us assume *k*_10_=*k*_21_=*k*_32_=*k*_43_=*k*, albeit scenarios with distinct rates are equally possible [9,10]. The closed form of *τ*_41_(*t*) is then given as

4.3 $$\tau_{41}(t)=\frac{k^{3}t^{2}}{2}{\text{e}}^{-kt},$$

and the one for *τ*_40_(*t*) is simply

4.4 $$\tau_{40}(t)=\frac{k^{4}t^{3}}{6}{\text{e}}^{-kt}=\frac{kt}{3}\tau_{41}(t),$$

for *t*≥0. A general formula can be found in [17].

The corresponding cumulative distribution functions (CDFs) are then given as

4.5 $$T_{41}(t)=1-{\text{e}}^{-kt}\sum_{j=0}^{2}\frac{k^{2-j}}{(2-j)!}t^{2-j}=1-{\text{e}}^{-kt}\left(\frac{k^{2}}{2}t^{2}+kt+1\right)$$

and

4.6 $$\begin{array}{rl} T_{40}(t) & =1-{\text{e}}^{-kt}\sum_{j=0}^{3}\frac{k^{3-j}}{(3-j)!}t^{3-j}=1-{\text{e}}^{-kt}\left(\frac{k^{3}}{6}t^{3}+\frac{k^{2}}{2}t^{2}+kt+1\right)\\ & =T_{41}(t)-{\text{e}}^{-kt}\frac{k^{3}}{6}t^{3}=T_{41}(t)-\frac{1}{k}\tau_{40}(t). \end{array}$$

With ***X***(*t*)=(*X*_1_(*t*),*X*_2_(*t*)), where the first entry corresponds to the number of walkers in state 0 and the second entry refers to walkers in state 4, the corresponding DCME of the first abridgment scheme becomes

4.7 $$\begin{array}{rl} \frac{\partial }{\partial t}P(\text{X},t) & =-k\text{X}(1)P(\text{X},t)+k(\text{X}(1)+1)P(\text{X}+{\left(1,0\right)}^{T},t)-\sum_{{\text{X}}_{i}\in I(\text{X})}\int_{0}^{t}k{\text{X}}_{i}(1)\tau_{41}(s)P(\text{X},t;{\text{X}}_{i},t-s)\;\text{d}s\\ & \;+\sum_{{\text{X}}_{i}\in I(\text{X})}\int_{0}^{t}k{\text{X}}_{i}(1)\tau_{41}(s)P(\text{X}-{\left(0,1\right)}^{T},t;{\text{X}}_{i},t-\tau )\;\text{d}s. \end{array}$$

Both integral terms include a propensity that depends on a previous state ***X***_*i*_, thus preventing any simplification of the integral terms.

Now, we will ask a different question: can one find a direct, closed-form solution for this system?

For a single walker in state 0 at time 0, i.e. *P*((1,0)^*T*^,0)=1, we know that the probability of the walker being in state 4 is

4.8 $$P({\left(0,1\right)}^{T},t)=T_{40}(t).$$

Also, since the delayed reaction is consuming, we know that

4.9 $$P({\left(1,0\right)}^{T},t)={\text{e}}^{-kt}=1-F(t),$$

where *F*(*t*)=1−e^−*kt*^ is the CDF of the exponential distribution with parameter *k*.

Note there is a third possible state, namely (0,0)^*T*^. The time evolution of its probability is

4.10 $$P({\left(0,0\right)}^{T},t)=1-(1-F(t))-T_{40}(t)=F(t)-T_{40}(t).$$

More generally, the probability of finding *m* out of *N* walkers in state 4 is

4.11 $$P({\left(*,m\right)}^{T},t)=\frac{N!}{m!(N-m)!}{\left(T_{40}(t)\right)}^{m}{\left(1-T_{40}(t)\right)}^{N-m}.$$

Likewise, the probability of finding *n* out of *N* walkers in state 0 is

4.12 $$P({\left(n,*\right)}^{T},t)=\frac{N!}{n!(N-n)!}{\left(1-F(t)\right)}^{n}{\left(F(t)\right)}^{N-n}.$$

In addition, we know that the probability of finding *l* of *N* walkers neither in state 0 nor in state 4 is

4.13 $$P({\left(*,*\right)}^{T},t)=\frac{N!}{l!(N-l)!}{\left(F(t)-T_{40}(t)\right)}^{l}{\left(1-F(t)+T_{40}(t)\right)}^{N-l},$$

where (*,*)^*T*^ denotes the set of states {(*n*,*m*)^T^|*l*+*m*+*n*=*N*}.

Putting all arguments together we arrive at a solution for *P*((*n*,*m*)^T^,*t*), for any state (*n*,*m*)^T^ with *n*+*m*≤*N*. Namely, it is simply the multinomial

4.14 $$\begin{array}{rl} P({\left(n,m\right)}^{T},t) & =\frac{N!}{n!m!(N-(n+m))!}{\left(1-F(t)\right)}^{n}{\left(T_{40}(t)\right)}^{m}\\ & {\left(F(t)-T_{40}(t)\right)}^{N-(n+m)}. \end{array}$$

The latter is not surprising, since the work in [5] suggests such a distribution as the solution of the CME for the unabridged system. Here, however, we obtain a similar distribution by using a delay. The three so-called ‘event probabilities’ of our multinomial distribution are 1−*F*(*t*), *T*_40_, and *F*(*t*)−*T*_40_(*t*), i.e. the time-dependent probabilities of a walker to be in state 0, state 4, or neither state 0 nor state 4 (the walker is on its way), respectively.

**Figure 2.**
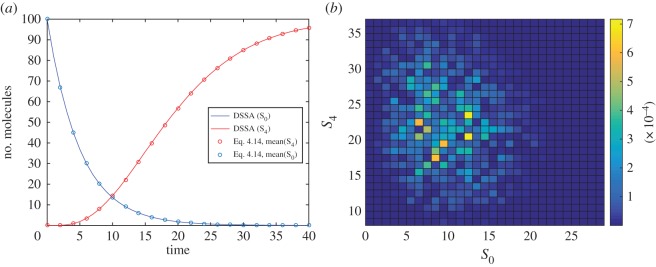
(*a*) Time evolution of the mean number of molecules in states 0 and 4, obtained from DSSA simulations (solid lines) or the mean of the multinomial distribution in equation (4.14). Here, *k*=0.2 and the system is in state (100,0) at time *t*=0. (*b*) Absolute error of the DSSA-derived probability distribution with respect to the closed-form solution (equation (4.14)) at time *t*=12. The error is at most approximately 7×10^−4^. Statistics were obtained from 10^5^ DSSA simulations.

Figure 2*a* shows a comparison of the result of the mean numbers of molecules in states 0 and 4 over time, as obtained from the multinomial distribution in equation (4.14) and DSSA simulations. Figure 2*b* presents the absolute error between the calculated (equation (4.14)) and statistically derived (DSSA) probability distributions at time *t*=12.

Importantly, the specific form of the delay distribution (here obtained from three consecutive linear unidirectional reactions with identical rate) does not change the form of the solution distribution. In other words, the solution of the DCME for a single unimolecular reaction *S*_0_→*S*_1_ with rate *k* and delay PDF *τ*(*t*) is always a multinomial distribution with event probabilities 1−*F*(*t*), *T*(*t*) and *F*(*t*)−*T*(*t*) with *T*(*t*) being the CDF of *k*e^−kt^*τ(*t*). One can readily see that if the delay is zero, i.e. *τ*(*t*)=*δ*_0_, then *T*(*t*)=1−e^−*kt*^=*F*(*t*) and the last event probability *F*(*t*)−*T*(*t*) drops out. This makes sense since without delays the number of molecules of *S*_0_ and *S*_1_ has to be constant at all times (*m*+*n*=*N*). Then, as expected, the resulting probability distribution simply becomes the binomial distribution with parameters *N* and e^−*kt*^. In the case that the delay is constant, *τ*(*t*)=*δ*_*s*_=*δ*(*t*−*s*) for a constant time delay *s*. Then *k*e^−kt^*δ_*s*_=*k*e^−*k*(*t*−*s*)^ for *t*≥*s* (and 0 otherwise). The CDF of this distribution is then 1−e^−*k*(*t*−*s*)^ for *t*≥*s* (and 0 otherwise). The rest follows as described above.

In summary, in this section we described a closed-form solution of the DCME for a single-delayed reaction in terms of its delay distribution. This is the first closed-form solution described for a delayed stochastic system. An ansatz for further analysis of this DCME can be found in the electronic supplementary material, S1.

## 5. Direct solution, without simulations, of the delay chemical master equation

As mentioned in the previous sections, the DCME had only been studied by generating trajectories with a DSSA. In this section, we show how it may be possible to numerically solve the DCME, without deriving an analytical closed-form solution, and without resorting to simulating independent trajectories by a DSSA. To do so, we will consider an mRNA maturation example.

**Figure 3.**
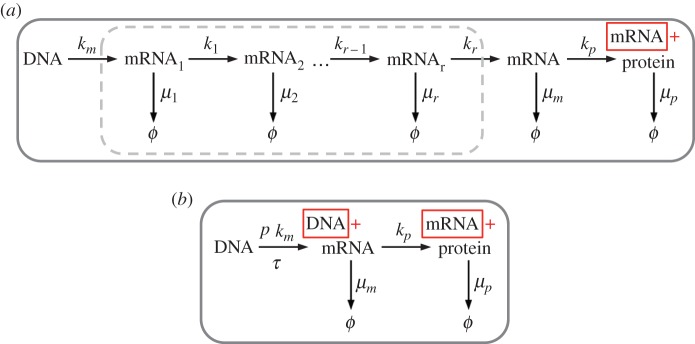
(*a*) Original model of mRNA maturation [18]. (*b*) Abridged model.

We start with the model presented in [18], with *r* intermediate chemical steps, illustrated in figure 3*a*. The associated reaction network can be exactly lumped by the system shown in figure 3*b* [9,10].

The abridged system consists of one delayed reaction for mRNA production (reaction R1: DNA→DNA+mRNA) and three non-delayed reactions (R2: mRNA→mRNA+Protein, R3: mRNA→0, R4: Protein→0). The delayed reaction has a rate that is equal to the production rate *k*_*m*_ of mRNA_1_ times the probability *p* for arriving at state ‘mRNA’ (as opposed to being degraded) and a delay distribution that describes a random walker's first-passage time to state ‘mRNA’ after starting in state ‘mRNA_1_’.

In the original model in [18], protein production is represented by a single reaction. Biochemically, this is not very accurate, as translation is a complex, time-consuming process. Likewise, any kind of feedback mechanisms in the form of transcription factor(s) binding to DNA are not considered here. Usually, such mechanisms are phenomenologically introduced into models in the form of Hill-type kinetics of the mRNA production reaction, or mechanistically in the form of one or more additional species representing DNA-bound states together with associated binding/unbinding reactions. In our abridged model we follow [18]. Specifically, we do not consider feedbacks and include protein production only as a non-delayed reaction. However, including Hill-type reactions, transcription factor binding, or a delayed translation reaction does not pose any problems with respect to DSSA simulations, but it is likely to complicate the derivation of a DCME that is solvable (see below).

Let us start by assuming *μ*=*μ*_1_=⋯=*μ*_*r*_ and *k*=*k*_1_=⋯=*k*_*r*_. This will simplify the derivation of a DCME without any loss of generality. As a first step, we obtain the delay distribution and compare SSA and DSSA simulations of the two reaction schemes. The PDF of the delay distribution is obtained as the convolution of exponential distributions with parameters corresponding to the absolute values of the eigenvalues of the associated transition matrix (4.5). Assuming the matrix rows/columns correspond to the states ‘mRNA_1_’, ‘mRNA_2_’, … ‘mRNA_*r*_’, this transition matrix has entries *k* on its subdiagonal and −*β*=−(*μ*+*k*) on its diagonal. Hence, we obtain one eigenvalue of multiplicity *r* with absolute value *β*.

In this case, the PDF of the delay distribution has the following closed form (4.13)

5.1 $$f(t)=\frac{\beta^{r}t^{r-1}}{(r-1)!}{\text{e}}^{-\beta t},$$

for *t*≥0.

The CDF is then given as

5.2 $$F(t)=1-{\text{e}}^{-\beta t}\sum_{k=0}^{r-1}\frac{\beta^{r-1-k}}{(r-1-k)!}t^{r-1-k}.$$

Let ***X***=(−,***X***(2),***X***(3))^T^ be a state of our abridged system, where ***X***(2) is the number of mRNA and ***X***(3) the number of protein molecules in the system. Note here ***X***(1) serves as a placeholder and has no meaning. ***I***(***X***) denotes the set of all possible system states in the past from which state ***X*** is able to follow via a chain of chemical reactions.

The corresponding DCME is

5.3 $$\begin{array}{rl} \frac{\partial }{\partial t}P(\text{X},t) & =-(k_{p}\text{X}(2)+\mu_{m}\text{X}(2)+\mu_{p}\text{X}(3))P(\text{X},t)+k_{p}\text{X}(2)P(\text{X}-{\left(0,0,1\right)}^{T},t)\\ & \;+\mu_{m}(\text{X}(2)+1)P(\text{X}+{\left(0,1,0\right)}^{T},t)+\mu_{p}(\text{X}(3)+1)P(\text{X}+{\left(0,0,1\right)}^{T},t)\\ & \;-\sum_{{\text{X}}_{i}\in I(\text{X})}\int_{0}^{t}k_{m}^{\prime }f(\tau )P(\text{X},t;{\text{X}}_{i},t-\tau )\;\text{d}\tau \\ & \;+\sum_{{\text{X}}_{i}\in I(\text{X})}\int_{0}^{t}k_{m}^{\prime }f(\tau )P(\text{X}-{\left(0,1,0\right)}^{T},t;{\text{X}}_{i},t-\tau )\;\text{d}\tau , \end{array}$$

where we use *k*′_*m*_=*k*_*m*_ *p*, and *p* is the probability of arriving at state ‘mRNA’ (as opposed to being degraded). As it has been previously shown [10], we can calculate the probability *p* as

5.4 $$p=\frac{\prod_{i=1}^{r}k_{i}}{\prod_{k=1}^{r}\bar{\lambda_{k}}},$$

where the $\bar{\lambda_{k}}$ are the absolute values of the eigenvalues of the *r*×*r* transition matrix describing the mRNA maturation; here $\bar{\lambda_{k}}=\beta$. This corresponds to a part of the original model in [18], illustrated in figure 3*a*, surrounded by a grey-dashed line.

In general, the DCME is not solvable given the *joint* probabilities are usually unknown. However, the following simplification has been previously proposed for DCMEs with constant delays [19]: if the time delays are large and there is a relatively large number of reactions in the time interval [*t*−*τ*,*t*) then the coupling of the system states at *t* and *t*−*τ* is weak and one can use the following approximation: *P*(***X***,*t*;***X***_*i*_,*t*−*τ*)≈*P*(***X***,*t*)*P*(***X***_*i*_,*t*−*τ*).

In our particular example, the triggering of the delayed reaction is fully independent of the occurrences of other reactions and of the state ***X***_*i*_ at the time of triggering. This allows us to simplify the joint probability terms without loss of accuracy. The probability that the system is in state ***X*** at time *t* while a delayed reaction gets updated in [*t*,*t*+d*t*) that has been previously triggered at time *t*−*τ* with delay *τ* is then:

5.5 $$\sum_{{\text{X}}_{i}\in I(\text{X})}k_{m}^{\prime }P(\text{X},t;{\text{X}}_{i},t-\tau )=k_{m}^{\prime }\sum_{{\text{X}}_{i}\in I(\text{X})}P(\text{X},t|{\text{X}}_{i},t-\tau )P({\text{X}}_{i},t-\tau )=k_{m}^{\prime }P(\text{X},t).$$

Replacing the constant delay with our delay distribution we obtain

5.6 $$k_{m}^{\prime }P(\text{X},t)\int_{0}^{t}f(\tau )\text{d}\tau =k_{m}^{\prime }P(\text{X},t)F(t).$$

Likewise, for a constant delay *τ*, the probability that the system is one update of *R*_1_ away from state ***X*** (namely in ***X***−(0,1,0)^T^) and the due update yields state ***X*** in [*t*,*t*+d*t*) is,

5.7 $$\sum_{{\text{X}}_{i}\in I(\text{X})}k_{m}^{\prime }P(\text{X}-{\left(0,1,0\right)}^{T},t;{\text{X}}_{i},t-\tau )=k_{m}^{\prime }P(\text{X}-{\left(0,1,0\right)}^{T},t),$$

while for a distributed delay it is

5.8 $$k_{m}^{\prime }P(\text{X}-{\left(0,1,0\right)}^{T},t)\int_{0}^{t}f(\tau )\;\;\text{d}\tau =k_{m}^{\prime }P(\text{X}-{\left(0,1,0\right)}^{T},t)F(t).$$

In summary, we can write down the DCME of the abridged reaction system as

5.9 $$\begin{array}{rl} \frac{\partial }{\partial t}P(\text{X},t) & =-(k_{p}\text{X}(2)+\mu_{m}\text{X}(2)+\mu_{p}\text{X}(3))P(\text{X},t)+k_{p}\text{X}(2)P(\text{X}-{\left(0,0,1\right)}^{T},t)\\ & \;+\mu_{m}(\text{X}(2)+1)P(\text{X}+{\left(0,1,0\right)}^{T},t)+\mu_{p}(\text{X}(3)+1)P(\text{X}+{\left(0,0,1\right)}^{T},t)\\ & \;+k_{m}^{\prime }(P(\text{X}-{\left(0,1,0\right)}^{T},t)-P(\text{X},t))F(t). \end{array}$$

It is important to note that this simplification is possible due to the propensity function of the delayed reaction being constant. This is only the case if none of the reactions in the system (including the delayed reaction) effectively change the number of reactants of the delayed reaction, nor its kinetic function.

So, how do we solve this homogeneous system of linear first-order ODEs with *variable* coefficients? Unfortunately, our system is not purely governed by monomolecular reactions but also by a catalytic reaction (R2). Otherwise we could apply the theory from [5] and obtain a closed solution. In our case this approach is not possible. Instead, we employ the finite state projection method [6] and solve the finite number of ODEs numerically. For the latter, we use Matlab's ODE solver.

**Figure 4.**
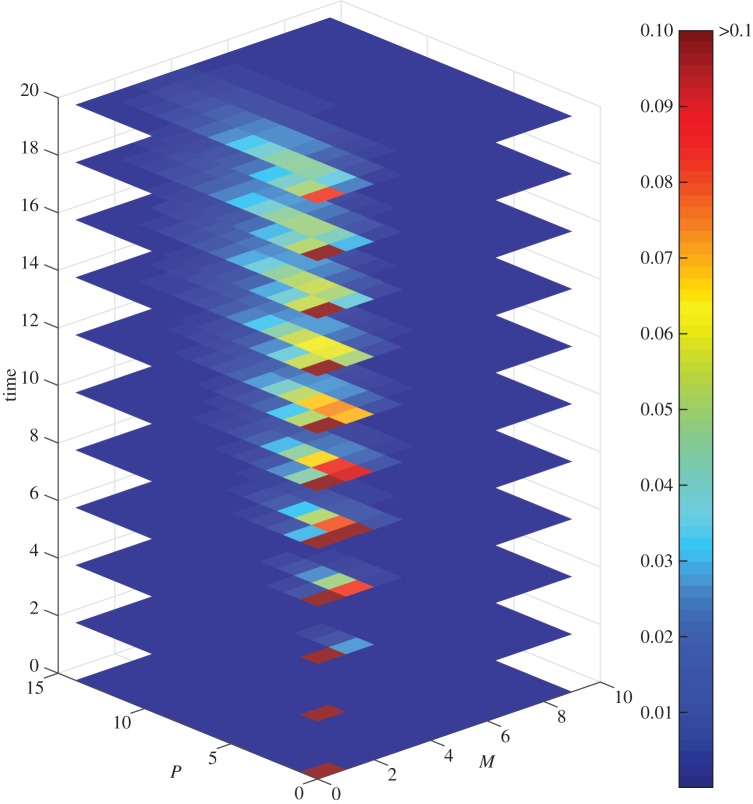
Time evolution of the DCME solution with initial state (*M*,*P*)=(0,0). Different slices correspond to different time points. Each rectangle represents a state (*M*,*P*). Its colour refers to the probability of observing the system in this state at that time.

**Figure 5.**
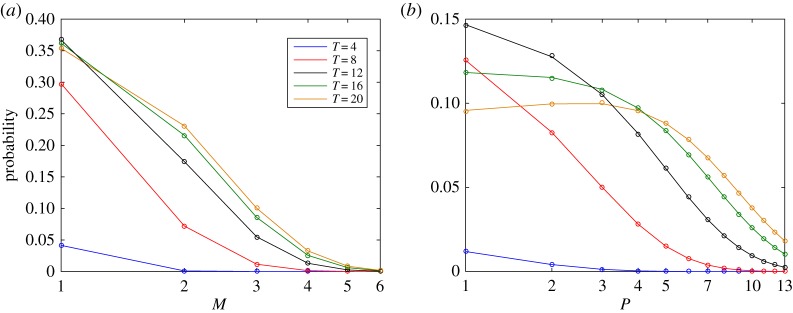
Probability distributions (with logarithmic scale on the *x*-axis) for the number of (*a*) *M* and (*b*) *P* molecules at time points *T*=4,8,12,16 and 20. DCME solutions are drawn as solid lines. Probabilities obtained from 1 000 000 SSA runs are shown as dots. Both solutions match perfectly.

Figure 4 presents the time evolution of the DCME solution of our system for *r*=7, *μ*=*μ*_*m*_=*μ*_*p*_=0.2, and *k*=*k*_*m*_=*k*_*p*_=1, when starting at state (*M*,*P*)=(0,0) and assuming a single DNA. Figure 5 shows a comparison of the DCMC direct numerical solution against statistics obtained from independent SSA simulations. The number of molecules *M* and *P* were limited to values between [0, 9] and [0, 14], respectively. For this state space, the error of the FSP is around 0.1%, and results show a remarkably good fit.

**Figure 6.**
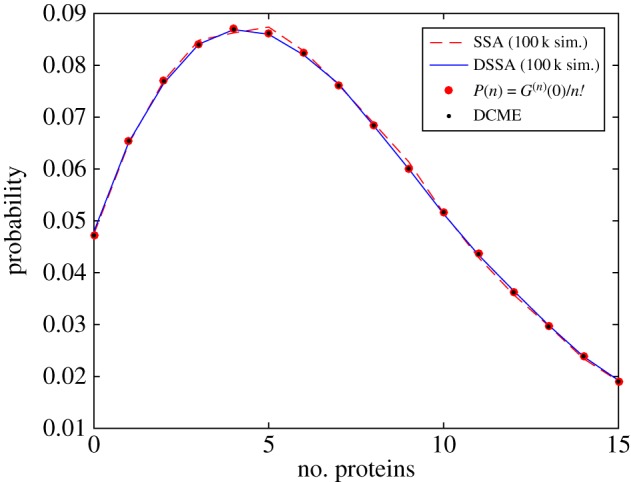
Comparison of DCME solution (black dots), *P*(*n*) using the generating function *G* (red circles) and histograms obtained from SSA (red dashed line) or DSSA (blue line) simulations after 70 time units. For the same number of simulations, and when recording system states every 0.1 time units, the DSSA is roughly a factor of 2.3× faster than the SSA.

Lastly, in figure 6 we show a comparison of the PDF for the number of proteins in the system at steady state at time *t*=70 time units. The data is shown as a histogram obtained from 100 000 SSA simulations, by solving the DCME at very large t, and using the following steady-state generating function

$$G(z)=\lim_{N\to \infty }\text{exp}\left(N\left\{{{\;}_{1}F}_{1}\left[\frac{k_{\mathrm{eq}}/N}{\mu_{p}};\frac{\mu_{m}}{\mu_{p}};\frac{k_{p}}{\mu_{p}}(z-1)\right]-1\right\}\right),$$

derived in [18]. Here, *k*_eq_=*k*_*m*_(*k*_1_/(*k*_1_+*μ*_1_)⋯(*k*_*r*_/(*k*_*r*_+*μ*_*r*_)) and _1_*F*_1_ is a generalized hypergeometric function. Additionally, we show the histogram from 100 000 DSSA simulations of the abridged system using the previously calculated delay distribution.

Of importance, this is the first time a DCME has been directly, numerically solved. Note that the DCME in equation (5.9) is simply a CME with a single time-varying factor. Interestingly, as shown in the electronic supplementary material, S2, its solution can also be obtained by using an SSA for time-varying rates.

## 6. Conclusion

Calculating exact solutions of CMEs is possible only when the system is either linear or the state space is reasonably small. This task becomes even more ambitious when introducing reactions with constant or distributed delays. In contrast to the CME, not a single example could be found in the literature where the DCME is explicitly calculated (or approximately calculated other than by using a DSSA sampling algorithm).

In this paper, we solve the DCME directly for two very simple reaction schemes: (i) a single unimolecular delayed reaction and (ii) a simple model of unregulated, delayed transcription and non-delayed translation. For the former example, we obtained a delay distribution by lumping a chain of linear, unidirectional reactions. By consequence, the analytic solution of the DCME was a multinomial distribution where the delay appeared in two out of three event probabilities. We show that this result generalizes to all kinds of delay distributions.

In the second example we do not simulate, but solve the DCME numerically. This is possible without relying on any approximations of joint probabilities as the propensity of the delayed reaction is independent of the current system state. Interestingly, the DCME in this particular case can be simplified to a CME with a time-varying rate representing the effect of the delay. This allowed us to simulate the system also with an SSA for time-varying rates (cf. electronic supplementary material, S2) yielding results close to that of DSSA simulations and our numerical solution of the DCME. However, it remains unclear to which extent there is a dualism between delays and time-varying rates. So far, there does not seem to be a way to show this holds for more general scenarios involving other delayed reactions—in particular those that have consuming/state-dependent delayed reactions, where simplifications of the DCME do not seem possible and, hence, the delay cannot be simply transformed.

For the other direction—namely, where we have a time-varying rate and want it to be treated as a delay—we have the (minimal) requirement that the time-varying factor has the properties of a CDF, i.e. it is non-decreasing, right-continuous, and has limits 0 and 1 when the time approaches 0 and infinity, respectively. The time-varying rate can then be expressed as an integral of its corresponding PDF, which describes the delay distribution. However, even in this limited case, it is not obvious how to derive a proper delayed reaction representation of a reaction with a time-varying rate. Let us illustrate this with an example: assume a reaction A→B with time-varying rate *kF*(*t*) that meets the requirements above, i.e. *F*(*t*) is a CDF. The CME consists of two terms, both have *kF*(*t*) as a factor, one is *kF*(*t*)***X***(1)*P*(***X***,*t*). A valid DCME will have terms *P*(***X***,*t*;***X***_*i*_,*t*−*τ*) for a delay *τ* and previous states ***X***_*i*_ (unless they vanish as in our example above) but there is no natural or intuitive way to introduce these. In this context, note that for a constant delay the time-varying factor simply vanishes—however, this only implies that a constant delay would never suffice to mimic a time-varying rate.

Lastly, for nonlinear reaction systems, very efficient CME solvers have been proposed that approximate the true solution with acceptable accuracy by using finite state projection methods [6,20]. Moment-closure approximations [21–25] provide an alternative to sampling methods and numerical solutions of CMEs. For the latter, the otherwise infinite set of moment equations for the number of molecules is ‘closed’ by setting the moments above a certain order equal to zero. However, it remains to be seen whether similar methods are applicable to the stochastic reaction kinetics with delays and, if so, what their limitations are.

## Supplementary Material

Appendix / Supplementary Material

## Data accessibility

All data are included in this article and derived from numerical solutions and stochastic simulations as described in the main text.
