# Supplementary material for: Delay chemical master equation: direct and closed-form solutions
Source: Proc Math Phys Eng Sci. 2015 Jul 8;471(2179):20150049. doi: 10.1098/rspa.2015.0049 (PMC4528653; doi:10.1098/rspa.2015.0049)
Supplement: Appendix / Supplementary Material [file rspa20150049supp1.pdf]

Appendix / Supplementary Material:

S1: DCME reconstruction for the example in Section 4

From our solution in Eq. (18), we can also reconstruct a DCME, for example for $N = 2$:

$$\frac{\partial}{\partial t} P_{20} = -2k\, P_{20}$$

$$\frac{\partial}{\partial t} P_{10} = 2k\, P_{20}\; - kP_{10} - \mathbf{2}\,(\mathbf{1} - \mathbf{F})\, \boldsymbol{\tau}_{\mathbf{40}}$$

$$\frac{\partial}{\partial t} P_{11} = -k\, P_{11} \underline{+\, 2\,(1 - F)\, \tau_{40}}$$

$$\frac{\partial}{\partial t} P_{00} = k\, P_{10}\; - \mathbf{2}\,(\mathbf{F} - \mathbf{T}_{\mathbf{40}})\, \boldsymbol{\tau}_{\mathbf{40}}$$

$$\frac{\partial}{\partial t} P_{01} = \; k\, P_{11} \underline{+2\,(\mathrm{F} - \mathrm{T}_{40})\, \tau_{40}} - \mathbf{2}\, \mathbf{T}_{\mathbf{40}}\, \boldsymbol{\tau}_{\mathbf{40}}$$

$$\frac{\partial}{\partial t} P_{02} = \underline{2\, \mathrm{T}_{40}\, \tau_{40}}.$$

Here we used P_{nm} instead of $P((n,m)^T, t)$, T_{40} instead of $\mathrm{T}_{40}(t)$, τ_{40} instead of $\tau_{40}(t)$ and F instead of $\mathrm{F}(t)$. The terms that are neither **bold** nor <u>underlined</u> correspond to the first two terms in the DCME:

$$-k\, \boldsymbol{X}(1)\, P(\boldsymbol{X}, t)\; + k\, (\boldsymbol{X}(1) + 1)\, P(\boldsymbol{X} + (1,0)^T, t)\,.$$

The **bold** and <u>underlined</u> terms correspond to the third and fourth term in the DCME, respectively:

$$-\sum_{\boldsymbol{X}_i \in \boldsymbol{I}(\boldsymbol{X})} \int_{\mathbf{0}}^{\boldsymbol{t}} \boldsymbol{k}\, \boldsymbol{X}_i(\mathbf{1})\, \boldsymbol{\tau}_{\mathbf{41}}(\boldsymbol{\tau})\, \boldsymbol{P}(\boldsymbol{X}, \boldsymbol{t}; \boldsymbol{X}_i, \boldsymbol{t} - \boldsymbol{\tau})\, \boldsymbol{d\tau}$$

$$\underline{+ \sum_{\boldsymbol{X}_i \in \boldsymbol{I}(\boldsymbol{X})} \int_0^t k\, \boldsymbol{X}_i(1)\, \tau_{41}(\tau)\, P(\boldsymbol{X} - (0,1)^T, t; \boldsymbol{X}_i, t - \tau)\, d\tau}$$

By comparing **bold**/<u>underlined</u> terms in the system of ODEs (derived from the solution) with the corresponding delay terms in the DCME, one can intuit the link between the general DCME terms and those in the ODEs. However, it is far from obvious how this may yield an alternative formulation of the DCME to the one reconstructed from the closed solution, in particular a computable DCME.

S2: DCMEs as CMEs with a time-varying rates

The DCME derived for the abridged model in Section 5 turns out to be a CME with a time varying factor. Thus, to simulate this chemical reaction system we can also use an SSA for time varying rates (tvSSA), such as [14, 26] instead of using a DSSA. A comparison of simulation results is shown in Figure S2. Note that simulations with the tvSSA were about three orders of magnitude more computationally expensive than comparable SSA simulations.

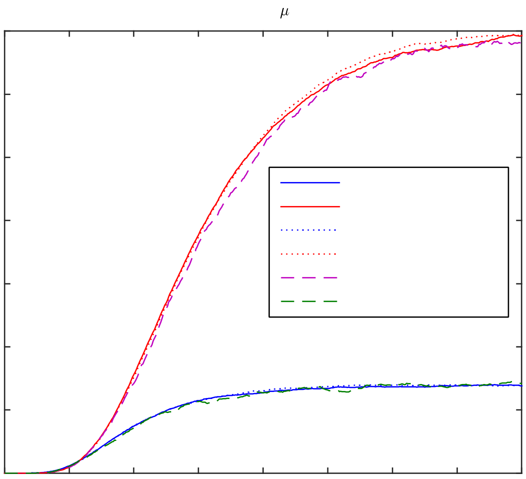

Figure S2: Comparison of SSA, DSSA and time-varying (tv)SSA. All three approaches match. Note that for SSA and DSSA simulations we used a 10 times larger sample number (10,000) than for tvSSA. Now, considering the smaller sample number for all three methods and simulating over 40 time units with states being recorded every 1 time unit, we observe that the DSSA is roughly 3.5x faster than the SSA, which in turn is three orders of magnitude faster than the tvSSA.
